# Efficacy and Safety of Filgotinib in Rheumatoid Arthritis Patients Aged over and under 65 Years (ENANTIA-65)

**DOI:** 10.3390/jpm14070712

**Published:** 2024-07-02

**Authors:** Maurizio Benucci, Marco Bardelli, Massimiliano Cazzato, Francesca Bartoli, Arianna Damiani, Francesca Li Gobbi, Francesca Bandinelli, Anna Panaccione, Luca Di Cato, Laura Niccoli, Bruno Frediani, Marta Mosca, Serena Guiducci, Fabrizio Cantini

**Affiliations:** 1 Rheumatology Unit, S. Giovanni di Dio Hospital, 50143 Florence, Italy; francesca.ligobbi@uslcentro.toscana.it (F.L.G.); francesca.bandinelli@uslcentro.toscana.it (F.B.); 2 Rheumatology Unit, Department of Medicine, Surgery and Neurosciences, University of Siena, 53100 Siena, Italy; marco.bardelli@ao-siena.toscana.it (M.B.); bruno.frediani@unisi.it (B.F.); 3 Unit of Rheumatology, University Hospital of Pisa, 56126 Pisa, Italy; massimiliano.cazzato@unipi.it (M.C.); marta.mosca@unipi.it (M.M.); 4 Department of Clinical and Experimental Medicine, University of Florence, 50139 Florence, Italy; francesca.bartoli@aou-careggi.toscana.it (F.B.); arianna.damiani@unifi.it (A.D.); serena.guiducci@unifi.it (S.G.); 5 Internal Medicine and Rheumatology Unit, Santa Maria General Hospital, 05100 Terni, Italy; anna.panaccione@tiscali.it (A.P.); l.dicato@aospterni.it (L.D.C.); 6 Division of Rheumatology, Prato Hospital, 59100 Prato, Italy; laura.niccoli@uslcentro.toscana.it (L.N.); fabrizio.cantini@uslcentro.toscana.it (F.C.)

**Keywords:** Filgotinib, lipid profile, rheumatoid arthritis, real life

## Abstract

Background: According to recent data, the age of patients could represent an important risk factor for MACE (major cardiovascular events), cancer, and VTE (venous thromboembolism) during treatment with JAK inhibitors in rheumatoid arthritis. We decided to analyze the population involved in the ReLiFiRa study by identifying two groups of patients: 65 years or more and less than 65 years of age, evaluating the efficacy and tolerability of 200 mg of Filgotinib daily. Methods: Of the 120 ReLiFiRa patients, 54 were younger than 65 years old and 66 patients were 65 years old or older. The data of efficacy and tolerability of treatment with FIL 200 mg daily for 6 months were evaluated. Results: After six months of treatment, FIL was effective in both age groups. In both groups, the median values of steroid DAS28, CDAI, ERS, PCR, tender joints, swollen joints, VAS, HAQ, PGA patients, and PGA physicians were reduced with a statistically significant difference comparing these values with the baseline values. The difference in age did not impact the effectiveness of the drug. The lipid profile data also did not demonstrate significant differences between the two age groups; however, the comparison between younger vs. older patients’ populations regarding the total cholesterol/HDL ratio and LDL/HDL ratio shows a statistically significant difference: total cholesterol/HDL 3.4 (2.12–3.66) vs. 3.64 (3.36–4.13) *p* = 0.0004, LDL/HDL 1.9 (0.98–2.25) vs. 2.41 (2.04–2.73) *p* = 0.0002. There are no differences regarding the atherogenic index (LDL-C/HDL-C) and coronary risk index (TC/HDL-C) compared to baseline. Conclusions: After six months of treatment with FIL, the older population group showed a higher level of LDL and a lower level of HDL compared to younger patients. The atherogenic index and coronary risk index are higher in patients aged ≥ 65 years, but interestingly, there were no differences when comparing the 6-month data to baseline values. This condition highlights the impact of typical risk factors that act independently of treatment with Filgotinib.

## 1. Introduction

### 1.1. Filgotinib Clinical Development

The first-generation Janus kinase inhibitors (JAKis) tofacitinib and baricitinib were approved for the treatment of rheumatoid arthritis before the role of the individual Janus kinase was clarified. The impact of this treatment on the transduction process regarding this entire family of tyrosine kinases is good control of inflammation, but it can also represent a risk of potential hematopoietic and metabolic disorders. Consequently, it was thought that more selective JAK1 inhibition could allow the same clinical efficacy as pan-JAK but with a better safety profile since the role of JAK-2 on erythropoietin, leptin, and thrombopoietin is known [1]. Filgotinib (FIL) is a selective JAK inhibitor with a 30-fold greater preference for JAK1 over JAK2 [2]. Clinical trial programs have evaluated FIL in patients with moderate to severe active rheumatoid arthritis (RA) across three phase 2 studies (DARWIN 1–3) and four phase 3 studies (FINCH 1–4). The DARWIN 3 and FINCH 1 studies evaluated the use of FIL in combination with MTX in patients with an inadequate response (IR) compared to methotrexate (MTX), according to the second-line therapy recommended in the EULAR treatment algorithm. FINCH2 evaluated the use of FIL in combination with conventional synthetic disease-modifying antirheumatic drugs (csDMARDs) in patients with failure or intolerance regarding previous biological disease-modifying antirheumatic drugs (bDMARDs), according to the third-line therapy recommended in the EULAR treatment algorithm. The FINCH 3 study evaluated the use of FIL in patients naïve to MTX. At present, a long-term extension called FINCH 4 exists, but no data are yet available [3]. Filgotinib achieved the endpoint in each study, without safety concerns.

### 1.2. Tofacitinib and Safety Concerns

In January 2022, the New England Journal of Medicine published the results of the ORAL Surveillance study, a randomized, open-label, noninferiority, post-authorization, safety end-point trial involving patients with active rheumatoid arthritis despite methotrexate treatment who were 50 years of age or older and had at least one additional cardiovascular risk factor. Patients were randomized into three groups: 1455 patients receiving tofacitinib (TOFA) at a dose of 5 mg twice daily, 1456 patients on tofacitinib at a dose of 10 mg twice daily, and 1451 patients receiving a TNF inhibitor. The analyses demonstrated that the incidence of MACE and cancer was higher with combined doses of tofacitinib (3.4% (98 patients) and 4.2% (122 patients), respectively) compared to a TNF inhibitor (2.5% (37 patients) and 2.9% (42 patients)). Hazard ratios were 1.33 (95% confidence interval (CI), 0.91–1.94) for MACE and 1.48 (95% CI, 1.04–2.09) for tumors [4]. The direct consequence was a warning from the Committee for Medicinal Products for Human Use (CHMP), which is the European Medicines Agency (EMA), involving not only TOFA but also baricitinib (BARI), upadacitinib (UPA), and FIL [5]. However, after the publication of the ORAL surveillance study, the real-world data deriving from the STAR-RA study [6], a post-hoc analysis from the same study concerning patients younger than 65 years with a low risk of atherosclerotic cardiovascular disease (ASCVD) <5% [7], did not confirm the risk of MACE and cancer, nor did the data from the TOFA clinical program regarding rheumatoid arthritis [8]. Recently, a pharmacovigilance study evaluated the relationship between thromboembolic events and JAKis. These data, provided by the Food and Drug Administration’s Adverse Event Reporting System, provided new safety signals on thromboembolic events for JAKis [9].

### 1.3. From the Trials to Real Life

It is very important to highlight that one of the most important risk factors of MACE and VTE in RA patients is an uncontrolled disease, so it becomes fundamental that the treatment is effective for a long time. In the real-life data, it is possible to find evidence of the similar effectiveness of TOFA and bDMARD [10,11], but two large studies have suggested better drug persistence of TOFAs compared to Tumor Necrosis Factors (TNFs), at least after the failure of the first biological treatment, and in this case, there are also no concerns about safety [12,13]. Real-world evidence remains limited for BARI because there are only small studies where efficacy and safety data are compared with TOFA and the results are inconclusive because of the number of confounding factors. [14,15]. A recent retrospective study evaluated RA patients who received a JAKi from four care centers in Milan. Six hundred and eighty-five patients were included and received BARI (48%), TOFA (31%), UPA (14%), or FIL (7%), while 47% of patients had been treated utilizing JAKi as a first-line treatment (before biologic). Over a total of 1137 patient-years, there was 1 stroke and 123 (18%) adverse events of special concern (AESI), including 3 deaths, all due to serious infections. A higher frequency of adverse events of special concern (23%) was observed in patients with higher cardiovascular risk [16]. In a recent real-life observational study, ReLiFiRa, conducted on 120 patients from rheumatology centers in Italy located in Tuscany and Umbria regions, we demonstrated that FIL therapy in patients with rheumatoid arthritis is safe and effective. The study population was identified as difficult to treat due to the high proportion of patients who had failed prior conventional and b-DMARD therapy [17]. In another retrospective study involving 194 patients treated with the JAK inhibitor, 57.9% were classified as ineligible for treatment according to the EMA restrictions. The most frequent reason for ineligibility was an increased risk of MACE (70.2%), followed by age >65 years (34.2%), smoking (30.7%), and an increased risk of VTE (20.2%) or malignant tumors (7%). The use of the Expanded Risk Score (ERS-RA) reduced the rate of patients carrying an increased CV risk to 18.6% (*p* < 0.001 compared to ORALSURV), leading to 46.4% of patients overall being ineligible [18]. Data from 182 patients with RA treated with JAKis in three Italy centers (Tuscany) were analyzed retrospectively. In total, 78.6% had at least one risk factor, including age ≥ 65 years, obesity, smoking, hypertension, dyslipidemia, hyperuricemia, diabetes, previous VTE, cancer, and severe mobility impairment. Seventy adverse events were observed (28/100 patient-years), including fifteen serious events (6/100 patients/year). No significant differences were observed after stratification by JAKis molecules. The presence of risk factors and the cumulative number of risk factors, as well as age ≥ 65 years, can predict the occurrence of adverse events [19]. According to recent data, the age of patients could represent an important risk factor for MACE, cancer, and VTE, so we decided to analyze the population involved in the ReLiFiRa study [17] by identifying two groups of patients—those aged 65 years or more and less than 65 years.

## 2. Materials and Methods

### 2.1. Participants

We evaluated the Efficacy and Safety of Filgotinib in Rheumatoid Arthritis Patients older and younger than 65 years (ENANTIA-65). The data from the study of the 120 ReLiFiRa patients were separated into two groups—one group with 54 patients who were under 65 years of age and one group with 66 patients who were 65 years old or older.

Table 1 shows the baseline characteristics. Clinimetric disease activity features and many laboratory parameters were not different comparing the two patient groups, except for the following data: diabetic patients 4.60% < 65 vs. years old 24.50% ≥ 65 years old (*p* = 0.0006), hypertension 22.70% < 65 years old vs. 67.90% ≥ 65 years old (*p* = 0.0007), statin use 6.80% < 65 years old vs. 16.98% ≥ 65 years old (*p* = 0.05), median steroid dosage 5 (0–8) < 65 years old vs. 4 (0–5) ≥ 65 years old (*p* = 0.043), median total cholesterol mg/dL 182.5 (159.5–203) < 65 years old vs. 204.5 (183.25–211) ≥ 65 years old (*p* = 0.03), median LDL-C mg/dL 104.3 (92–129.5) < 65 years old vs. 120.5 (104.25–134) ≥ 65 years old (*p* = 0.043)), and median triglycerides mg/dL 95 (80.5–120) < 65 years old vs. 114 (100–131.75) ≥ 65 years old (*p* = 0.0097).

### 2.2. Statistical Analysis

Descriptive statistics were used for the basic features of the population and, because the data did not show a normal distribution, we used the median and Inter Quartile Range (IQR). For comparison, the Wilcoxon test for paired samples and the Mann–Whitney test for independent samples were used. To compare the incidences, we used the Fisher exact test, and for correlations, we used the Correlation Coefficient. A *p*-value less than 0.05 was considered statistically significant. Statistical analysis was performed using © 2023 MedCalc Software Version 22.021 Ltd., Acacialaan 22, 8400 Ostend Belgium.

## 3. Results

### Efficacy and Safety of Filgotinib: Differences in RA Patients Younger and Older Than 65 Years

In Table 2, we report the data after 6 months of treatment with Filgotinib in patients aged <65 years and >65 years old.

The percentage of patients treated with Methotrexate was 19.50% in the younger group and 25.50% in the older population, both of which were less than the percentage found at baseline for both groups.

Regarding the use of statins, the percentage after 6 months was 11.40% of patients in the younger group and 28.12% for the older group, showing a numerical increase compared to the baseline for both populations.

After 6 months of treatment, there were no differences regarding steroid dosage, showing zero as the median value for both cohorts.

Regarding clinical data, the DAS28 value, the percentage of patients in DAS28-LDA, and the percentage of patients in DAS28 remission were the same in the two cohorts, as well as for CDAI. There were also no differences regarding PGA patients, PGA physicians, VAS, HAQ, or swollen joint values. The difference that we found was related to the Tender Joint count (0 (0–2) vs. 2 (1–2) *p* = 0.0032), where the older population showed a greater number of joints still involved. Also, regarding the inflammation indices ESR and CRP, there were no differences when comparing the two cohorts after six months of treatment with FIL.

Regarding the metabolic picture, comparing the younger and older groups, there were no differences in body weight, BMI, Total cholesterol, and triglycerides.

There were differences in LDL-C mg/dL (110.5 (92.95–129.5) vs. 121 (111–145) *p* = 0.038), with higher values in the older patients; HDL-C mg/dL (60 (48.75–91) vs. 56 (45–59.25) *p* = 0.047), with higher values in the younger patients; and markers of cardiovascular risk, where Total cholesterol/HDL (ratio) (3.4 (2.12–3.66) vs. 3.64 (3.36–4.13) *p* = 0.0004) and LDL/HDL (ratio) (1.9 (0.98–2.25) vs. 2.41 (2.04–2.73) *p* = 0.0002) were higher in the older cohort. There were no differences in Creatinine, Aspartate aminotransferase, Alanine aminotransferase, or Hemoglobin values. At the end of observation, we had not observed MACE, Cancer, VTE, Herpes zoster infection, or opportunistic infections in either cohort. Table 3 shows the cardiovascular risk factor profile at baseline vs. after 6 months of therapy in patients younger and older than 65 years.

## 4. Discussion

### 4.1. Analysis of the Results

Recent concerns about the use of JAKi in patients over 65 years of age and in the presence of cardiovascular risk factors have stimulated numerous retrospective analyses in order to understand if this kind of problem really exists and if it involves all the drugs belonging to the JAKi group. Our retrospective observational study analyzed data from 120 patients with difficult-to-treat rheumatoid arthritis divided into two groups based on age: patients younger than 65 years and patients 65 years of age or older. In the 65-year-old population, there was a higher prevalence of diabetes and hypertension, while in the younger age group, there were more smokers. There were no differences in disease duration, and we did not observe a correlation between disease duration and age (r = 0.0264). These data show that patients aged ≥65 years had a late onset of the disease. In the ≥65-year cohort, there were more patients treated with statins and the median steroid dose was slightly lower. Despite the use of statins, in the ≥65-year cohort, the median concentration of total and LDL cholesterol was higher than in younger subjects, as was the concentration of triglycerides. The differences regarding the atherogenic index (LDL-C/HDL-C) and the coronary risk index (TC/HDL-C) are likely due to the impact of age-related risk factors because the data did not change after six months of treatment with FIL. No cases of MACE, cancer, VTE, HZ infection, or opportunistic infections were observed.

What interpretation should be given to these data based on literature evidence?

### 4.2. Literature Analysis

In previous studies [6,7], early interruptions of therapy have been shown due to the appearance of adverse events (14.4% for patients <65 years vs. 26.3% for patients aged 65 or more), often in a real-life setting [20]. In our study, there were no interruptions of treatment because, in the 6 months of treatment, we did not record adverse events or clinical worsening. In the younger group, there were more smokers and, as is known, cigarette smoking is an important risk factor for the development of RA [21] and is associated with an increase in disease activity [22] as well as a low response to therapy [23,24,25,26]. In our group, there were no differences between smokers and non-smokers regarding drug discontinuation and there were no differences between the two age groups. These data confirm the evidence from a phase 3 trial with FIL, where the efficacy was analyzed in nonsmokers, former smokers, and current smokers. FIL resulted in being effective in all subgroups analyzed regardless of smoking status. Furthermore, FIL appears to be superior to adalimumab in the current and former smoker groups with an inadequate MTX response [27]. In our observation, after six months of treatment with FIL, the older population shows a higher level of LDL and a lower level of HDL than younger patients, but without cardiovascular events. In the integrated safety analysis of FIL involving 4057 patients with 5493 patient-years of exposure (a median of 1.6 years and a maximum of 5.6 years) [28], the incidence rates of serious adverse events in the FIL group and the placebo group were the same. The incidence of MACE was similar for FIL 100 mg and 200 mg once daily, adalimumab, MTX, and placebo, as was the risk of VTE [29,30]. In the integrated safety analysis of FIL, increases in LDL-C and HDL-C were observed without an increase in the atherosclerotic index [28,29,30,31]. These data likely differentiate FIL from other JAK inhibitors. These types of drugs not only block cell signaling via JAK/STAT but also have cellular metabolic effects (including a decrease in mitochondrial membrane potential, mitochondrial mass, and ROSs and the inhibition of metabolic genes in synovial tissue) [32] and are able to modify systemic lipid metabolism. JAKis significantly increase HDL-C and LDL-C after treatment compared to baseline and other DMARDs, as demonstrated in randomized controlled trials [30,31,32], an effect that can be reversed with statin therapy [33]. JAKs also improve HDL function by increasing the activity of lecithin-cholesterol acyltransferase (LCAT; an enzyme that converts free cholesterol to cholesterol esters and supports cholesterol efflux to lipoproteins), increasing the efflux capacity of HDL [33,34]. Furthermore, effects such as alterations in the size and content of lipoproteins have been described [35,36,37]. A study using cultured human THP-1 macrophages evaluated the impact of TOFA on cellular cholesterol efflux and synthesis via radioisotopic methods and on cholesterol uptake by measuring the cholesterol content in cells with a fluorometric assay. TOFA significantly increased cholesterol efflux from macrophages, reduced cholesterol uptake from both normal and hypercholesterolemic sera, and reduced cholesterol synthesis [38]. In a recent systematic review with a meta-analysis of randomized controlled trials in rheumatoid arthritis from Pubmed, Medline, Embase, and the Cochrane Controlled Trials Register, data from 18 unique studies involving five approved JAKs and 6697 patients with rheumatoid arthritis (JAKi = 3341, placebo = 3356) showed an 8.11 mg/dL increase in HDL levels compared to baseline and a mean increase of 11.37 mg/dL in LDL levels compared to baseline, but the risk of cardiovascular disease did not differ significantly between patients who received JAK, the placebo, or other active agents [39]. In one study, one-year therapy with TOFA significantly increased TC, HDL, LDL, APOA, APOB, leptin, adipsin, and TSP-1 while significantly decreasing levels of Lp(a), chemerin, PON1, and MPO. T.G, lipid indices (TC/HDL and LDL/HDL), adiponectin, and resistin showed no significant changes. Numerous associations were found between lipids, adipokines, clinical markers, IMT, FMD, and PWV (*p* < 0.05) [40]. The better selectivity of FIL on JAK-1 in the absence of activity on JAK-2 may determine an absence of action on leptin, maintaining the feeling of satiety stability and could act on the lipid profile by indirectly keeping adiponectin active. Furthermore, the action on the lipid profile could be mediated by an inflammatory mechanism by IL-6 as for TOCI [41,42]. In one study, 27 healthy volunteers received single doses of atorvastatin (40 mg) and pravastatin (40 mg)/rosuvastatin (10 mg)—alone or with FIL (200 mg once daily for 11 days). Samples were collected via serial blood pharmacokinetics and safety was assessed. Pharmacokinetic parameters were evaluated using geometric least squares (GLSM) 90% confidence intervals (CIs) of the study treatment (coadministration of statins with FIL) compared to statin alone. The results indicated that FIL has no clinically significant effect on the exposure of atorvastatin, pravastatin, or rosuvastatin [43].

### 4.3. Real-Life Data

Our data are confirmed by another recent real-life study conducted on 126 patients in an Italian population in which only one cardiovascular event was recorded [44]. A recent collection of 246 rheumatoid arthritis patients (89% female, 57.6 ± 12.2 years) was treated with FIL, mostly as second-line (22%) or subsequent (43.9%) b/tsDMARD treatment. The survival rate of FIL was 84.5% at the 6-month follow-up and 75.8% at the 12-month follow-up. Fifty-one patients discontinued FIL during the follow-up, fifteen due to a lack of efficacy, eight due to a loss of efficacy, and eight due to adverse events (four recurrent infections, one herpes zoster virus, and three laboratory test abnormalities). No MACE or new onset tumors were reported [45]. Recent data from 7 clinical trials of 3691 patients who received FIL for a median (maximum) duration of 3.8 (8.3) years (12,541 PYE) showed no differences in cardiovascular events (MACE) and venous thromboembolism for doses of 100 and 200 mg of FIL [46]. In a recent real-life retrospective study about the use of JAK inhibitors, the LDL-C/HDL-C ratio in the FIL group did not change after one year of treatment, while the values increased, in a statistically significant manner, for the other three drugs [47].

## 5. Conclusions

Our data show that, when comparing patient features in RA patients younger than 65 years old and older than 65 years old, there are some important differences: in the older population, there is a greater prevalence of diabetes and hypertension, while in the younger group, there are more smokers. Regarding disease duration, there is no difference when comparing the two age groups and no correlation between age and disease duration, showing a later onset of disease in older patients. Despite the greater use of statins in the ≥65-year-old cohort, the median values of total and LDL cholesterol are higher than in younger subjects, as well as the triglyceride concentration. After six months of treatment with FIL, the older population shows a higher level of LDL and a lower level of HDL compared to younger patients. The atherogenic index and coronary risk index are higher in patients aged ≥ 65 years, but interestingly, there were no differences when comparing the 6-month data with the baseline values. This condition highlights the impact of typical risk factors, which act independently of treatment with Filgotinib.

## Figures and Tables

**Table 1:** Characteristics of patients younger and older than 65 years, at baseline.

Clinical Data (Median-IQR)	<65-Ys-Old (N 54, 45%)	>65-Ys-Old (N 66, 55%)	*p*-Value
Age (Years)	52 (40.7–58)	74 (70–76)	0.0001
Female sex	84.10%	86.80%	NS
Weight (kg)	66 (56–76)	68.5 (59.5–75)	NS
BMI	23.14 (19.57–25.95)	23.19 (21.59–28.12)	NS
Disease duration (years)	6 (3.7–10)	10 (5–14)	NS
DAS28	4.91 (4.23–5.92)	4.73 (4.36–5.33)	NS
CDAI	21.5 (18–22)	20 (18–23.75)	NS
Tender Joints	9 (5–12)	6 (5–10)	NS
Swollen Joints	6 (3.2–9)	4 (4–6)	NS
VAS pain	7 (7–8)	7 (3–8)	NS
HAQ	1 (1–1.5)	1 (1–1.25)	NS
PGA patients	70 (60–80)	65 (30–80)	NS
PGA physicians	70 (40–75)	60 (30–70)	NS
ESR mm/h	31 (17.25–43.25)	40 (20–56)	NS
CRP mg/dL	1.12 (0.6–2.02)	1.2 (0.5–1.8)	NS
ACPA positivity	75%	84.9%	NS
RF positivity	86.4%	96.22%	NS
ACPA-RF double positivity	72.70%	84.90%	NS
Previous HZ infection	6.80%	1.88%	NS
Previous VTE	0%	0%	NS
Previous MACE	2.27%	1.88%	NS
Smoking	27.30%	11.30%	NS
Hormone therapy	2.30%	0	NS
Diabetes	4.60%	24.50%	0.0006
Arterial hypertension	22.70%	67.90%	0.0007
Total cholesterol (mg/dL)	182.5 (159.5–203)	204.5 (183.25–211)	0.03
LDL (mg/dL)	104.3 (92–129.5)	120.5 (104.25–134)	0.043
HDL (mg/dL)	53 (47–65);	52.5 (45–57)	NS
Total cholesterol/HDL (mg/dL)	3.57 (2.76–3.94)	3.88 (3.61–4.1)	NS
LDL/HDL (mg/dL)	1.94 (1.48–2.42)	2.36 (2.06–2.62)	NS
Triglycerides (mg/dL)	95 (80.5–120)	114 (100–131.75)	0.0097
Hemoglobin (mg/dL)	12.5 (11.7–12.75)	12.6 (11.9–13.5)	NS
Creatinin (mg/dL)	0.65 (0.75–0.8)	0.8 (0.64–0.9	NS
AST (UI/L)	19 (13.5–19.5)	20 (15–25)	NS
ALT (UI/L)	15 (10.5–22)	18 (15–23)	NS
Statin	6.80%	16.98%	0.05
Steroid dosage (mg)	5 (0–8)	4 (0–5)	0.043
Methotrexate	29.50%	39.60%	NS
Leflunomide	4.60%	6%	NS
Sulfasalazine	6.80%	1.88%	NS
No biological failure	20.45%	26.40%	NS
1 biological failures	22.72%	18.86%	NS
2 biological failures	29.54%	26.40%	NS
3 biological failures	18.20%	15.14%	NS
4 biological failures	9.09%	11.32%	NS
5 biological failures	0%	1.88%	NS

**Table 2:** Characteristics of RA patients younger and older than 65 years at 6 months of FIL treatment from baseline.

Clinical Data 6 Months (Median-IQR)	<65-Ys-Old (N 54, 45%)	>65-Ys-Old (N 66, 55%)	*p*-Value
BMI	21.92 (20.31–23.93)	22.47 (21.48–23.68)	NS
DAS28	2.5 (2.08–3.1)	2.8 (2.33–3.1)	NS
CDAI	6 (4–10)	8 (0–10)	NS
LDA DAS28	32.40%	51.10%	NS
LDA CDAI	61.90%	51.28%	NS
Remission DAS28	54%	40%	NS
Remission CDAI	23.80%	28.20%	NS
Tender Joints (number)	0 (0–2)	2 (1–2)	0.0032
Swollen Joints (number)	1 (0–1)	0 (0–1)	NS
VAS	2 (1–4)	2 (1–3)	NS
HAQ	0.18 (0–0.5)	0.5 (0–0.75)	NS
PGA patient	20 (5–30)	20 (10–30)	NS
PGA physician	20 (10–30)	20 (10–20)	NS
ESR (mm/h)	12.5 (9.5–20.25)	15 (10–20.5)	NS
CRP (mg/dL)	0.25 (0.14–0.47)	0.3 (0.11–0.49)	NS
MACE (%)	0	0	NS
Cancer (%)	0	0	NS
VTE (%)	0	0	NS
Herpes zoster infection (%)	0	0	NS
Opportunistic infections (%)	0	0	NS
Total cholesterol (mg/dL)	206 (159–211)	201 (176–209)	NS
LDL (mg/dL)	110.5 (92.95–129.5)	121 (111–145)	0.038
HDL (mg/dL)	60 (48.75–91)	56 (45–59.25)	0.047
Triglycerides (mg/dL)	112 (89–121.5)	121 (108.5–132)	NS
Total cholesterol/HDL (mg/dL)	3.4 (2.12–3.66)	3.64 (3.36–4.13)	0.0004
LDL/HDL (mg/dL)	1.9 (0.98–2.25)	2.41 (2.04–2.73)	0.0002
Hemoglobin (mg/dL)	12.75 (11.97–13.4)	12.5 (12–13.4)	NS
Creatinine (mg/dL)	0.69 (0.65-0.84)	0.8 (0.62–0.9)	NS
Aspartate aminotrasferase (AST)	18 (14–20)	19 (14–23.5)	NS
Alanine aminotrasferasi (ALT)	15 (10–23)	19 (14–23.5)	NS
Methotrexate	19.50%	25.50%	NS
Leflunomide	5.70%	0%	NS
Sulfasalazine	5.70%	3.12%	NS
Statin	11.40%	28.12%	0.01
Average steroid dosage (mg)	0 (0–4)	0 (0–4)	NS
Patients without steroid	52.27%	54.71%	NS

**Table 3:** Risk factor profile in patients younger and older than 65 years at baseline and after 6 months of therapy.

Comparison of Cardiovascular Risk Factors Profile, Baseline vs. 6 Month (Median, IQR)	<65 Ys-Old	*p*	≥65 Ys-Old	*p*
Weight (kg)				
Baseline	66 (56–76)	NS	68.5 (59.5–75)	NS
6 Months	66 (59–74.5)		68 (58–73)	
BMI				
Baseline	23.14 (19.57–25.95)	NS	23.19 (21.59–28.12)	NS
6 Months	21.92 (20.31–23.93)		22.47 (21.48–23.68)	
Total cholesterol (mg/dL)				
Baseline	182.5 (159.5–203)	NS	204.5 (183.25–211)	NS
6 Months	206 (159–211)		201 (176–209)	
LDL (mg/dL)				
Baseline	104.3 (92–129.5)	NS	120.5 (104.25–134)	NS
6 Months	110.5 (92.95–129.5)		121 (111–145)	
HDL (mg/dL)				
Baseline	53 (47–65)	NS	52.5 (45–57)	NS
6 Months	60 (48.75–91)		56 (45–59.25)	
Triglycerides (mg/dL)				
Baseline	95 (80.5–120)	NS	114 (100–131.75)	NS
6 Months	112 (89–121.5)		121 (108.5–132)	
Total cholesterol/HDL (mg/dL)				
Baseline	3.57 (2.76–3.94)	NS	3.88 (3.61–4.1)	NS
6 Months	3.4 (2.12–3.66)		3.64 (3.36–4.13)	
LDL/HDL (mg/dL)				
Baseline	1.94 (1.48–2.42)	NS	2.36 (2.06–2.62)	NS
6 Months	1.9 (0.98–2.25)		2.41 (2.04–2.73)	

## Data Availability

The raw data supporting the conclusions of this article will be made available by the authors, without undue reservation.

## Abbreviations

RA	Rheumatoid arthritis
JAK	Janus kinase
STAT	signal transducers and activators of transcription
bDMARDs	biological disease-modifying antirheumatic drugs
csDMARDs	conventional syntetic disease-modifying antirheumatic drugs
JAKis	JAK inhibitors
ntj	number of tender joints
nsj	number of swollen joints
VAS	visual analogic scale
GA	global assessment
HAQ	health assessment questionnaire
PGA	patient global assessment
DAS28	Disease Activity Score
TC	total cholesterol
LDL-C	LDL-cholesterol
HDL-C	HDL-cholesterol
TG	triglycerides
MACE	major cardiovascular events
VTE	venous thromboembolism
TOFA	Tofacitinib
BARI	Baricitinib
FIL	Filgotinib
UPA	Upadacitinib
MTX	Methotrexate
ROS	reactive oxigen species
TNF	Tumor Necrosis Factor
Lp(a)	Lipoprotein a
APOA	Apolipoprotein-A
APOB	Apolipoprotein-B
TSP-1	Trombospondin-1
PON-1	Paraoxonase-1
MPO	Myeloperoxidase
IMT	Intima-Media-Thickness
FMD	flow-mediated dilatation
PWV	pulse wave velocity
